# The Nature of Protein Domain Evolution: Shaping the Interaction Network

**DOI:** 10.2174/138920210791616725

**Published:** 2010-08

**Authors:** Christoph P Bagowski, Wouter Bruins, Aartjan J.W te Velthuis

**Affiliations:** 1 German University Cairo, Faculty of Pharmacy and Biotechnology, New Cairo City, Egypt; 2 Institute of Biology, Leiden University, 2333 AL Leiden, The Netherlands; 3 Department of Medical Microbiology, Molecular Virology Laboratory, Leiden University Medical Center, Albinusdreef 2, 2333 ZA Leiden, The Netherlands; 4 Department of Bionanoscience, Delft University of Technology, Lorentzweg 1, 2628 CJ, Delft, The Netherlands

**Keywords:** Protein domain, PDZ domain, systems biology, superfamily, molecular evolution, interactome.

## Abstract

The proteomes that make up the collection of proteins in contemporary organisms evolved through recombination and duplication of a limited set of domains. These protein domains are essentially the main components of globular proteins and are the most principal level at which protein function and protein interactions can be understood. An important aspect of domain evolution is their atomic structure and biochemical function, which are both specified by the information in the amino acid sequence. Changes in this information may bring about new folds, functions and protein architectures. With the present and still increasing wealth of sequences and annotation data brought about by genomics, new evolutionary relationships are constantly being revealed, unknown structures modeled and phylogenies inferred. Such investigations not only help predict the function of newly discovered proteins, but also assist in mapping unforeseen pathways of evolution and reveal crucial, co-evolving inter- and intra-molecular interactions. In turn this will help us describe how protein domains shaped cellular interaction networks and the dynamics with which they are regulated in the cell. Additionally, these studies can be used for the design of new and optimized protein domains for therapy. In this review, we aim to describe the basic concepts of protein domain evolution and illustrate recent developments in molecular evolution that have provided valuable new insights in the field of comparative genomics and protein interaction networks.

## INTRODUCTION

The protein universe is the collection of proteins of all biological species that exist or have once existed on Earth [1]. Our sampling and understanding of it began over half a century ago, when the first peptide and protein sequences were determined by Sanger [2, 3] and, subsequently, the sequencing of RNA and DNA [4-6]. In the meantime, the genome projects of the last decade have uncovered an overwhelming amount of sequence data and researchers are now starting to address a series of fundamental questions that should shed light onto protein evolution processes [7-10]. For instance, how many gene encoding sequences are present in one genome? How many sequences are repetitive and are these sequences similar in the various organisms on Earth? Which genes were involved in the large scale genome duplications that we see in animals? 

A comparison of sequences for evolutionary insight is best achieved by looking at the structural and functional (sub)units of proteins, the protein domains. By convention, domains are defined as conserved, functionally independent protein sequences, which bind or process ligands using a core structural motif [11-13]. Examples of domain modes of actions in signaling cascades for instance, are to connect different components into a larger complex or to bind signaling-molecules [14, 15]. Protein domains can usually fold independently, likely due to their relatively limited size, and are well known to behave as independent genetic elements within genomes [16, 17]. The sum of these features makes protein domains readily identifiable from raw nucleotide and amino acid sequences and many protein family resources (e.g., Superfamily and SMART [see Table **1**]) indeed fully rely on such sequence similarity and motif identifications [18, 19].

## DOMAIN IDENTIFICATION, SEQUENCE ALIGNMENT AND PHYLOGENY

The algorithms that are used for domain identification are built around a set of simple assumptions that describe the process of evolution. In general, evolution is believed to form and mold genomes largely via three mechanisms, namely i) chemical changes through the incorporation of base analogs, the effects of radiation or random enzymatic errors by polymerases, ii) cellular repair processes that counter mutations, and iii) selection pressures that manifest themselves as the positive or negative influence that determines whether the mutation will be present in subsequent generations [20, 21]. By definition, each of these phenomena has its own rate, while their combined effect gives a certain probability for the change of one defined amino acid (or nucleotide) to another within a specific time interval.

**Fig. (1):**
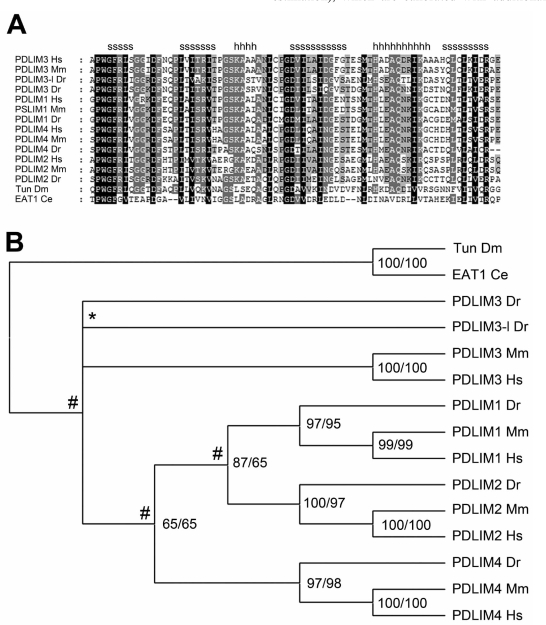
Example of sequence alignment and phylogeny. (**A**) This figure shows an example alignment of the PDZ domain with different shadings representing the amount of conservation (100, 75 or 50%) at a particular position in the sequence. (**B**) This tree is the phylogenetic presentation of the alignment in Fig. (**1A**). It was computed using Bayesian estimation and presents the best-supported topology for the alignment. Numbers indicate % support by the two methods used, while # indicates gene duplication events in the common ancestor and * marks a species-specific duplication event. For computational details, please see [42].

Although already informative in its own right, mutation data can be significantly different among species due to dissimilar metabolisms, generation times, population sizes, lifestyles, reproductive strategies, or the lack of apparent polymerase-dependent proofreading such as in positive-stranded RNA viruses [22-25]. Consequently, substitution rates need therefore be calculated to correctly compare two or more sequences and hunt uncharted genomes for comparable domains. Particularly this last strategy, using general rate matrices like BLOSOM and PAM, is an elegant example of how new protein functions can be discovered [26-30]. Fast algorithms for pair-wise alignments can be found in the Basic Local Alignment Search Tool (BLAST), whereas multiple sequence alignments (MSAs, Fig. **1A**) in which multiple sequences are compared simultaneously are commonly created with for example ClustalX and MUSCLE (see Table **1**) [31-34].

Close relatives, sharing an overall sequence identity above for example 50% and a set of functional properties, can also be grouped into families and subfamilies. In turn, these families share also evolutionary relationships with other domains and form together so-called domain superfamilies [18, 35]. Evolutionary distances between related domain sequences can easily be estimated from sequence alignments, provided that the correct rate assumptions are made. Subsequently, these can be used to compute the phylogenies of the domain that share an evolutionary history. These, often tree-like graphs (Fig. **1B**), depend heavily on rate variation models, such as molecular clocks or relaxed molecular clocks (e.g., Maximum Likelyhood and Bayesian estimation), which are calibrated with additional evidence such as fossils and may therefore also provide valuable information on aspects like divergence times and ancestral sequences [36-38]. Commonly used phylogenetic analysis strategies are listed in Table **1**. 

A limitation of all inferred phylogenetic data is that it is directly dependent on the alignment and less so on the programs used to build the phylogenetic tree [39]. One of the shortcomings of automated alignments may thus derive from the fact that they commonly employ a scoring and penalty procedure to find the best possible alignment, since these parameters vary from species to species [22, 23], as mentioned above. Careful inspection of alignments is therefore advisable, even though software has been developed that combines the alignment procedure and phylogenetic analysis iteratively in one single program [40]. 

## DOMAIN DIVERSIFICATION

Although sequence and phylogenetic analysis provide a relatively straightforward way for looking at domain divergence, comparison of solved protein structures has shown that protein tertiary organizations are much more conserved (>50%) than their primary sequence (>5%) [41]. For this reason, protein structures and their models provide significantly more insight into the relations of protein domains and how domain families diverged [16]. For example, the inactive guanylate kinase (GK) domain present in the MAGUK family was shown to originate from an active form of the GK domain residing in Ca2+ channel beta-subunits (CACNBs) through both sequence and structural comparison [42]. Furthermore, identification of functionally or structurally related amino acid sites in a fold sheds light on the complex, co-evolutionary dynamics that took place during selection [43]. 

As described above, the evolution of a protein domain is generally the result of a combination of a series of random mutations and a selection constraint imposed on function, i.e., the interaction with a ligand. The interaction between protein and ligand can be imagined as disturbances of the protein’s energy landscape, which in turn bring about specific, three-dimensional changes in the protein structure [44, 45]. Binding energies however, need not be smoothly distributed over the protein’s binding pocket as a limited number of amino acids may account for most of the free-energy change that occurs upon binding [45-47]. In these cases, new binding specificities (including loss of binding) may therefore arise through mutations at these hot spots. An example is a recent study of the PDZ domain in which it was shown that only a selected set of residues, and in particular the first residue of α-helix 2 (αB1), directly confers binding to a set of C-terminal peptides [48].

The folding of a domain is essentially based on a complex network of sequential inter-molecular interactions in time [49]. This has of course significant implications for domain integrity, particularly if one assumes that the core of a protein domain is and has to be largely structurally conserved. Indeed, even single mutations that arise in this area may easily derail the folding process, either because their free energy contribution influences residues in the direct vicinity or disturbs connections higher up in the intermolecular network [49]. It is therefore hypothesized that protein evolution took place at the periphery of the protein domain core, and that gradual changes via point mutations, insertions and deletions in surface loops brought about the evolutionary distance we see among proteins to date [21, 50-52]. 

However, distant sites also contribute to the thermodynamics of catalytic residues. This is achieved through a mechanism called energetic coupling, which is shaped by a continuous pathway of van der Waals interactions that ultimately influences residues at the binding site with similar efficiency as the thermodynamic hotspots [53, 54]. Indeed in such cases, evolutionary constraints are not placed on merely one amino acid in the binding pocket, but on two or more residues that can be shown to be statistically coupled in MSAs [54, 55]. In addition to contributions to binding, these principles also explain why the core of a domain structure will remain largely conserved, while at functionally related places residues can (rapidly) co-evolve with an overall neutral effect [56]. Of course, these aspects of co-evolution are also of practical consequence for structure prediction and rational drug design [43].

## DOMAIN DUPLICATION

Through selective mutation, protein domains have been the tools of evolution to create an enormous and diverse assembly of proteins from likely an initially relatively limited set of domains. The combined data in GenBank and other databases now covers over 200.000 species with at least 50 complete genomes and this greatly facilitates genome comparisons [57-59]. Following such extensive comparisons, currently > 1700 domain superfamilies are recognized in the recent release of the Structural Classification of Proteins (SCOP) [60] and it has become clear that many proteins consist of more than one domain [17, 61, 62]. Indeed, it has been estimated that at least 70% of the domains is duplicated in prokaryotes, whereas this number may even be higher in eukaryotes, likely reaching up to 90% [35].

**Fig. (2):**
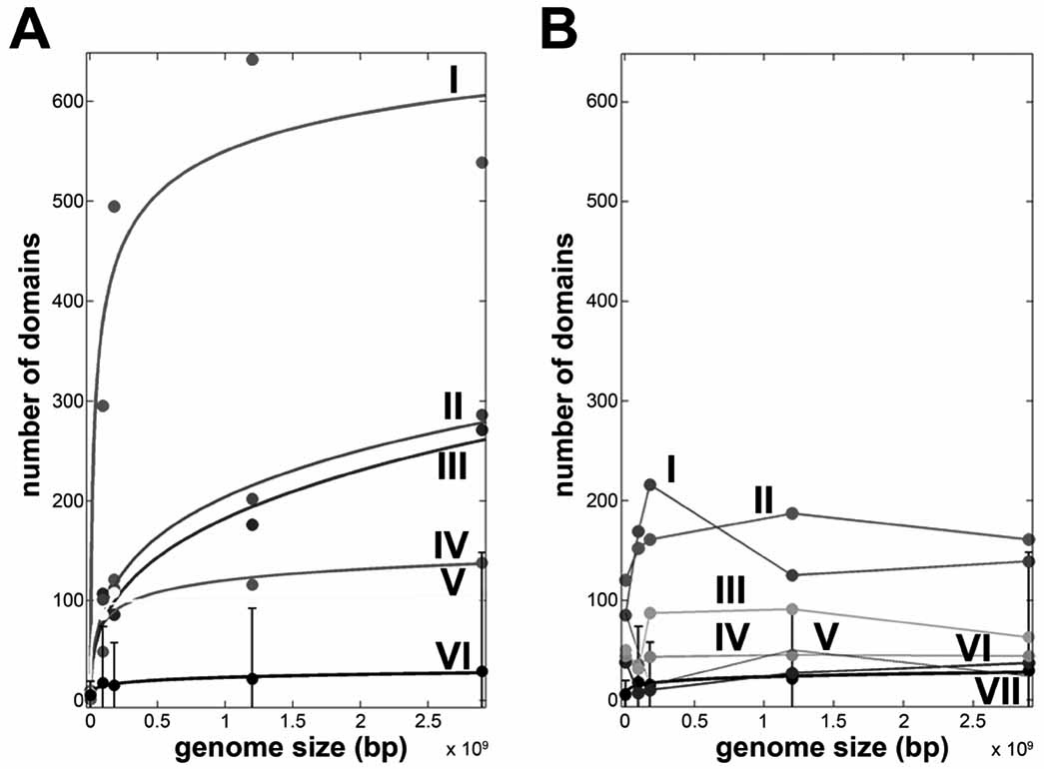
Selection on superfamily domain size. (**A**) Increase in superfamily domain size fitted to a power-law for kinase-like domains (I), Ankyrin-repeats (II), PDZ-like (III), voltage-gated potassium channels (IV), the catalytic domain of metalloproteases (V) and the average increase in superfamily size (VI). R^2^ value for each fit was at least 0.9. (**B**) Neutral or decreasing family sizes can be found for the MFS general substrate transporters (I), NAD(P)-binding Rossmann folds (II), Ribonucleases H (III), PLP-dependent transferases (IV), periplasmic binding proteins type II (V), ATPase domains of HSP90/topoisomerase II/histidine kinase-like folds (VI) and the average increase in superfamily size (VII) as in 2A.

There are various mechanisms through which protein domain or whole proteins may have been duplicated. On the largest scale, whole genome duplication such as those seen in the vertebrate genomes duplicated whole gene families, including postsynaptic proteins, hormone receptors and muscle proteins, and thereby dramatically increased the domain content and expanded networks [42, 63, 64]. On the other end of the scale, domains and proteins have been duplicated through genetic mechanisms like exon-shuffling, retrotranspositions, recombination and horizontal gene transfer [65-67]. Since the genetic forces, like exon-shuffling and genome duplication vary among species, the total number of domains and the types of domains present fluctuate per genome. Interestingly, comparative analyses of genomes have shown that the number of unique domains encoded in organisms is generally proportional to its genome size [60, 68]. Within genomes, the number of domains per gene, the so-called modularity, is related to genome size *via* a power-law, which is essentially the relation between the frequency *f* and an occurrence *x* raised by a scaling constant *k* (i.e., *f* (x) ~ x*^k^* ) [69, 70]. A similar correlation is found when the multi-domain architecture is compared to the number of cell types that is present in an organism, i.e., the organism complexity or when the number of domains in a abundant superfamily is plotted against genome size (Fig. **2**) [71, 72]. 

## DOMAIN SELECTION

Given the amount of domain duplication and apparent selection for specific multi-domain encoding genes in, for example, vertebrates, it may come as little surprise that not all domains have had the same tendency to recombine and distribute themselves over the genomes [68, 73]. In fact, some are highly abundant and can be found in many different multi-domain architectures, whereas others are abundant yet confined to a small sample of architectures or not abundant at all [68, 70]. Is there any significant correlation between the propensity to distribute and the functional roles domains have in cellular pathways?

Some of the most abundant domains can be found in association with cellular signaling cascades and have been shown to accumulate non-linearly in relation to the overall number of domains encoded or the genome size [70]. Additionally, the on-set of the exponential expansion of the number of abundant and highly recombining domains has been linked to the appearance of multicellularity [70]. A reoccurring theme among these abundant domains is the function of protein-protein interaction and it appears that particularly these, usually globular domains, have been particularly selected for in more complex organisms [70]. This positive relation is underlined by the association of these abundant domains with disease such as cancer and gene essentiality as the highly interacting proteins that they are part of have central places in cascades and need to orchestrate a high number of molecular connections [74, 75]. Their shape and coding regions, which usually lie within the boundaries of one or two exons, make them ideally suited for such a selection, since domains are most frequently gained through insertions at the N- or C-terminus and through exon shuffling [76-78].

From a mutational point of view, protein-protein interaction domains are different from other domains as well and this appears to be particularly true for the group of small, relatively promiscuous domains like SH3 and PDZ. These domains are promiscuous in the sense that they both tend to physically interact with a large number of ligands [79, 80] and are prone to move through the genome to recombine with many other domains. It has been found that particularly these domains evolve more slowly than non-promiscuous domains [70]. This likely stems from the fact that they are required to participate in many different interactions, which makes selection pressures more stringent and the appearance of the branches on phylogenetic trees relatively short and more difficult to assess when co-evolutionary data in terms of other domains in the same gene family or expression patterns is limited [42, 63]. Non-promiscuous domains on the other hand can quite easily evade the selection pressure by obtaining compensatory mutations either within themselves or their specific binding partner [70].

The overall phenomenon that the number of protein domains and their modularity increases as the genome expands has not been linked to a conclusive biological explanation yet. A rationale for the increase in interactions and functional subunits, however, may derive from the paradoxical absence of correlation between the number of genes encoded and organism complexity, the so-called G-value paradox [81]. There is indeed evidence that domains involved in the same functional pathway tend to converge in a single protein sequence, which would make pathways more controllable and reliable without the need for supplementary genes [73]. Additionally, the number of different arrangements found in higher eukaryotes is, given the vast scale of unique domains present, relatively limited. This in turn implies that evolutionary constraints have played an important role in selecting the right domain combinations and the right order from N- to C-terminus in multi-domain proteins [13, 82]. In fact, the ordering and co-occurrence of domains was demonstrated to hold enough evolutionary information to construct a tree of life similar to those based on canonical sequence data [70]. Furthermore, the increased use of alternative splicing and exon skipping in higher eukaryotes likely supplied a novel way of proteome diversification by restricting gene duplication and stimulating the formation of multi-domain proteins [83, 84]. In plants, however, the latter notion is not supported since both mono- and dicots show limited alternative splicing and a more extensive polyploidy [85-87].

## THE EVOLUTION OF DOMAIN INTERACTION NETWORKS

**Fig. (3):**
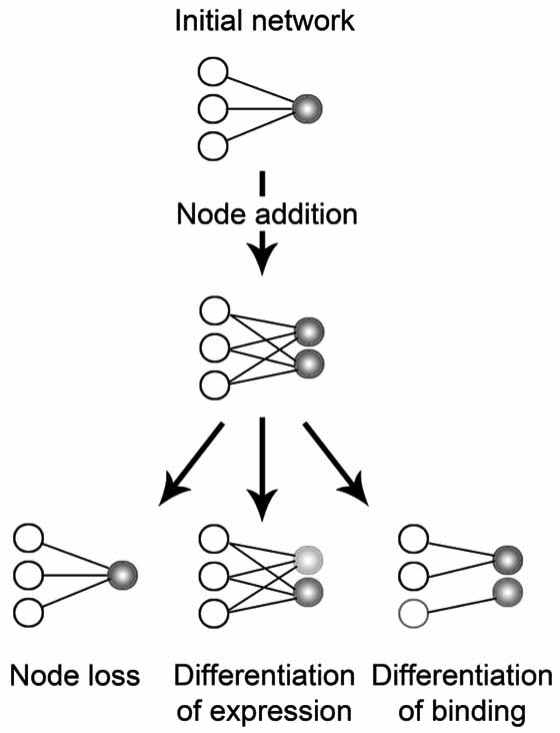
Evolutionary models for protein-protein interactions. The evolution of protein networks is tightly coupled to the addition or deletion of nodes. Additionally, events that introduce mutations in binding interfaces of proteins may result in the addition or loss of links in the network. Node addition may take place through *e.g.,* domain duplication or horizontal gene transfer, while rewiring of the network is mediated by point mutations, alternative splice variants and changes in gene expression patterns.

It is clear that some of the above characteristics are underappreciated in the phylogenetic analysis of linear amino acid sequences. Moreover, the effects of evolution extend even further than these aspects and entail transcriptional and translational regulation, intramolecular domain-domain interactions, gene modifications and post-translational protein modifications [88-96]. New methods are thus being developed to take into account that when sequences evolve, their close and distant functional relationships evolve in parallel. Correlations of mutations have already been found between residues of different proteins [97, 98] and compensating mutational changes at an interaction interface were shown to recover the instability of a complex [99]. These observations are evidence for the current evolutionary models for the protein-protein interaction (PPIs) networks that are being constructed through large-scale screens [100-102]. In these, a gene duplication or domain duplication (depending on the resolution of the network) implies the addition of a node, while the deletion of a gene or domain reduces the amount of links in the network (Fig. **3**). In the next step, extensive network rewiring may take place, driven by the effect of node addition or node loss in the network (i.e., the duplicability or essentiality of a domain/protein) and mutations in the domain-interaction interface [67, 74, 103-105]. 

Beyond mutations at the domain and protein level, regulation of protein expression provides another vital mechanism through which protein networks can evolve. Microarray studies are now well under way to map genome-wide expression levels of related and non-related genes under a variety of conditions [91, 94-96]. For example, transcriptional comparisons have investigated aging [106] and pathogenicity [107]. Unfortunately, given the highly variable nature of gene expression and the fact that different species may respond different to external stimuli, such comparisons can only be performed under strictly controlled research conditions. To date most studies have therefore focused on the embryogenesis, metamorphosis, sex-dependency and mutation rates of subspecies [94, 108-111]. Other studies have revealed valuable information on promoter types and duplication events [91-94].

To overcome the limitations mentioned in the previous paragraph, the analysis of co-expression data has been developed to supplement the direct comparison of individual gene expression changes [95]. In this procedure, a co-expression analysis of gene pairs within each species precedes the cross-comparison of the different organisms in the study. This approach thus primarily focuses on the similarity and differences of the orthologous genes within network, and is therefore ideally suited for the study of protein domain evolution and has already revealed that species-specific parts of an expression network resulted *via* a merge of conserved and newly evolved modules [95, 112, 113].

## CONCLUDING REMARKS

Finding evolutionary relationships protein domains is mostly based on orthology and thus commonly performed on best sequence matches. Identifying these and categorizing them depends largely on multiple sequence alignments and this will in most cases give good indications for function, fold and ultimately evolution. However, this approach usually discards apparent ambiguities that arise from species-specific variations (e.g., due to population size, metabolism or species-specific domain duplications or losses) and may therefore introduce significant biases [114]. Biases may also derive from the method of alignment, the rate variation model used to infer the phylogeny, and the sample size used to build the alignment [39, 40, 115]. Care should therefore be taken to not regard orthology as a one-to-one relationship, but as a family of homologous relations [91], to select for appropriate analysis methods [39, 115] and extend comparative data to protein interactions and expression profiles [91]. Indeed, as our wealth of biological information expands, our systems perspective will improve and provide us with an opportunity to reveal protein domain evolution at the level network organization and dynamics. Large-scale expression studies are beginning to show us evolutionary correlations between gene expression levels and timings [94, 106, 107, 112, 116], while others demonstrate spatial differences between paralogs or (partial) overlap between interaction partners [117-120]. Indeed, when we are able to map the spatiotemporal aspects of inter- and intra-molecular interactions we will begin to fully understand the versatile power of evolution that shaped the protein universe and life on Earth [118].

## FUNDING

AV is supported by The Netherlands Organization for Scientific Research (NWO) through Toptalent grant 021.001.037. 

## Figures and Tables

**Table 1.** List of Public Resources and Databases Relevant to Domain Analysis

Resource	URL
**Protein domain databases**	
Pfam	http://www.sanger.co.uk/Pfam/
Prosite	http://www.expasy.org/prosite/
SMART	http://smart.embl-heidelberg.de
Superfamily	http://supfam.mrc-lmb.cam.ac.uk/SUPERFAMILY/hmm.html
**Structural analysis**	
CATH	http://www.cathdb.info/latest/index.html
SCOP	http://scop.mrc-lmb.cam.ac.uk/scop/
SSM	http://www.ebi.ac.uk/msd-srv/ssm/
Swiss-MODEL	http://swissmodel.expasy.org/
**Alignment software**	
BLAST	http://www.ncbi.nlm.nih.gov/blast/Blast.cgi
ClustalW	http://www.ebi.ac.uk/Tools/clustalw2/
Muscle	http://www.ebi.ac.uk/muscle/
**Protein interaction**	
HPRD	http://www.hprd.org
MINT	http://mint.bio.uniroma2.it/mint/Welcome.do
STRING	http://string.embl.de/
**Phylogenetic analysis**	
MrBayes (*Bayesian*)	http://mrbayes.csit.fsu.edu/
PhyML (*Max. Likelihood*)	http://atgc.lirmm.fr/phyml/
PHYLIP (*various*)	http://evolution.genetics.washington.edu/phylip.html
CAPS (*residue coevolution*)	http://bioinf.gen.tcd.ie/~faresm/page11/page11.html
**Visualization**	
Pymol (*structural*)	http://pymol.sourceforge.net/
NJplot (*phylogeny*)	http://pbil.univ-lyon1.fr/software/njplot.html
DiepView (*structural*)	http://spdbv.vital-it.ch/
TreeView (*phylogeny*)	http://taxonomy.zoology.gla.ac.uk/rod/treeview.html
Visant (*protein interaction*)	http://visant.bu.edu/
**Sequence depositories**	
Ensembl (*genome projects*)	http://www.ensembl.org
PDB (*structures*)	http://www.rcsb.org/pdb/home/home.do
NCBI	http://www.ncbi.nlm.nih.gov/sites/gquery?itool=toolbar
UniProt	http://www.expasy.uniprot.org/
